# Assessing Diabetes and Factors Associated with Foregoing Medical Care among Persons with Diabetes: Disparities Facing American Indian/Alaska Native, Black, Hispanic, Low Income, and Southern Adults in the U.S. (2011–2015)

**DOI:** 10.3390/ijerph14050464

**Published:** 2017-04-26

**Authors:** Samuel D. Towne, Jane Bolin, Alva Ferdinand, Emily Joy Nicklett, Matthew Lee Smith, Marcia G. Ory

**Affiliations:** 1 Department of Health Promotion and Community Health Sciences, School of Public Health, Texas A&M University, College Station, TX 77846, USA; health@uga.edu (M.L.S.); mory@sph.tamhsc.edu (M.G.O.); 2 Department of Health Policy and Management, School of Public Health, Texas A&M University, College Station, TX 77846, USA; JBolin@sph.tamhsc.edu (J.B.); ferdinand@sph.tamhsc.edu (A.F.); 3 School of Social Work, University of Michigan, Ann Arbor, MI 48109, USA; enicklet@umich.edu; 4 Institute of Gerontology, Department of Health Promotion and Behavior, College of Public Health, University of Georgia, Athens, GA 30602, USA

**Keywords:** health disparities, environmental and social predictors, place-based disparities

## Abstract

*Objective*: Identify individual- and place-based factors associated with diagnosed diabetes and forgone medical care among those diagnosed with diabetes. *Background*: Diabetes affects millions of individuals globally. In the U.S. alone the prevalence rate of diagnosed diabetes has more than doubled over the past 20 years (4.2% in 1994 to 10% in 2014). *Methods*: The Behavioral Risk Factor Surveillance System (2011–2015) was used to identify factors associated with self-reported diabetes diagnoses (ever diagnosed) among U.S. adults. Logistic regression modeled: (1) the likelihood of having diabetes; (2) the likelihood of forgone medical care among those with diabetes, given appropriate medical care has been linked to preventing complications associated with diabetes. *Results*: Rates of diabetes remained relatively stable from 2011 to 2015. The likelihood of diabetes was higher (*p* < 0.01) among racial and ethnic minority groups, men, those with lower incomes and those with lower education. Place-based disparities indicating a higher likelihood of having a diagnosis of diabetes were found for those living in rural areas (urban versus rural, unadjusted OR = 0.844–0.908; *p* < 0.01) and those living in the South (North, Midwest, and Western/Pacific regions versus the South, unadjusted OR = 0.794–0.889; *p* < 0.01). Similar results were found with forgone medical care among those diagnosed with diabetes being more likely in the South (North, Midwest, and Western/Pacific regions versus the South, unadjusted OR = 0.542–0.819). In fully-adjusted analyses, the prevalence of diabetes and forgone medical care among those diagnosed with diabetes was higher for those with lower incomes, from several racial/ethnic minority groups, and in the South versus most other regions. *Conclusions*: Identifying at-risk groups informs targets for prevention and assists efforts to address chronic disease self-management among those already diagnosed with diabetes.

## 1. Introduction

Diabetes is a significant public health burden with over 29 million (21.0 million diagnosed; 8.1 million undiagnosed) individuals of all ages affected in the United States (U.S.) [1,2]. Type 2 diabetes accounts for approximately 95% of all cases of diabetes [2] and as such national estimates of diabetes represent Type 2 diabetes in the vast majority of cases. Additionally, 86 million American adults are estimated to have prediabetes [2]. Prediabetes includes those with higher than normal blood glucose levels, yet lower than that for a diagnosis of diabetes [3]. Thus, the burden associated with diabetes is a major societal concern. 

In the U.S., the overall crude percentage of adults *diagnosed* with diabetes grew from 4.2% in 1994 to 10% in 2014 [4]. Uncontrolled diabetes is a potentially debilitating disease [5] due to its impact on other organs and the cardiovascular system. Further, those with diabetes have twice the risk of age-adjusted mortality as compared to those without diabetes [1,6]. Almost all diabetes is *largely* preventable with 90–95% of all diagnosed diabetes attributable to type 2 diabetes [7] and modifiable factors including healthy lifestyle behaviors including, among other things, achieving adequate levels of physical activity and eating a healthful diet [8]. Thus, there is significant opportunity to ameliorate the fact that millions suffer from diabetes related complications [3]. Further, while prevention of diabetes itself is the most desired option, prevention of complications associated diabetes is critical given complications can be avoided with proper planning and management (e.g., monitoring blood glucose, blood pressure) [1]. At the same time, access to safe opportunities to engage in physical activity or access to healthy foods is an obvious compliment to these individual-level behaviors [9].

### 1.1. Geospatial and Social Determinants of Health

Type 2 diabetes is associated with many sociodemographic factors such as age [2], race/ethnicity [2], and socioeconomic status [10]. There are also documented geographic variations in the prevalence rates of diabetes, with rates in the Southern U.S. higher than the national average [11]. The World Health Organization’s Framework for Action on the Social Determinants of Health [12] notes that multiple determinants of individual-level social characteristics (e.g., race/ethnicity, income, education) and a structural characteristics (e.g., place-based factors) predict health and health-related outcomes. Thus, this framework can be helpful to identify individual-level and structural factors associated with diabetes and related health outcomes.

In addition to older adults [2], racial and ethnic minority groups are disproportionately affected by type 2 diabetes [13]. For example, the likelihood of diabetes is higher among Black or African American, Hispanics/Latino, and American Indian individuals as compared to non-Hispanic White individuals [14]. Socioeconomic factors are independently associated with type 2 diabetes onset among racial/ethnic minorities [15]. For example, Black or African American individuals experiencing household or neighborhood-level poverty are at heightened risk of developing diabetes [14]. American Indians and Alaska Native individuals served by the Indian Health Service had a higher rate (over 14%) of diagnosed diabetes than any other racial or ethnic group in 2010 [1]. Further, American Indian individuals have been shown to be more than three times as likely to die from diabetes than White individuals [16]. Diabetes rates were also higher among non-Hispanic Black or African American (nearly 13%) and Hispanic (nearly 12%) adults compared to non-Hispanic Asian (over 8%) and non-Hispanic White (just over 7%) adults [1]. Further, the complications associated with diabetes are related to higher likelihoods of morbidity and mortality [17]. Thus, certain racial/ethnic groups are disproportionately more likely to develop diabetes. Further, diabetes-related complications can be largely controllable (e.g., monitoring blood glucose levels, blood pressure, cholesterol) [17] suggesting that access to proper screening may be critical. 

Disparities in the rates of diabetes have also been shown to be associated with geospatial factors such as residing in rural (versus urban) areas throughout the U.S., where the relative rates of diabetes were higher in rural areas for the overall U.S. population, and within racial and ethnic groups among White and Black or African American adults [18]. However, rates were lower in rural areas for Hispanic adults relative to their urban Hispanic counterparts [18]. In addition to rates of diabetes, there are major gaps in access to health care providers for treatment and management of diabetes [19]. This has been shown in rural regions relative to urban areas [19] throughout the U.S. As such, rurality must be considered in terms of access and utilization of medical care among potentially at-risk groups (e.g., those with diabetes). In summary, both rurality and race/ethnicity play major roles in assessing the impact of diabetes and access to care throughout the U.S. for those with a diagnosis of diabetes.

### 1.2. Objective

To inform targeted intervention strategies, it is critical to monitor trends in diabetes prevalence over time, factors associated with diabetes, and access to care among those with diabetes. Given that diabetes-related disparities are present across race, ethnicity, poverty, and place (e.g., rurality), there is a need to better understand factors associated with diabetes and associated barriers to treatment—such as cost or scarcity of providers and services—across time and place. This need is consistent with calls for research identifying disparities among racial and ethnic minority groups over time [13]. Further, there is a need to identify both individual and structural factors associated with health-related outcomes, as laid out in the Framework for Action on the Social Determinants of Health [12]. Documenting changes or trends over time can help to identify consistent trends or new gaps in access as they arise. Given the socioeconomic gradient in diabetes prevalence and care has been observed, this study investigates whether any changes in this gradient occurred over time. Our objective was to describe trends in prevalence of diabetes and care for diabetes by socioeconomic and demographic (e.g., ethnic) characteristics. Specifically, we aimed to identify individual and community factors associated with: (1) diabetes (i.e., among those having ever been told by a health care provider one was diagnosed with diabetes); and (2) unmet need, or forgoing medical care deemed recommended or necessary due to cost in the past 12 months among those with diabetes. 

## 2. Materials and Methods

### 2.1. Data

Based on self-reported survey data, the Behavioral Risk Factor Surveillance System (BRFSS) provides nationally representative estimates for the U.S. non-institutionalized adult population. Data include individual-level health-related outcomes. We used the 5-year period between 2011 and 2015 to conduct this study. The sample size of each year varied, with 506,467 for 2011, 475,687 for 2012, 491,773 for 2013, 464,664 for 2014, and 441,456 for 2015.

### 2.2. Dependent Variables

#### 2.2.1. Dependent Variable 1

Diagnosed diabetes was assessed as the major outcome in the study both independently as an outcome and as a way to subset forgone medical care among those with a diagnosis of diabetes. Individuals were asked if they have ever been told they had diabetes stemming from the question “*Has a doctor, nurse, or other health professional EVER told you that you had any of the following?*”. Responses indicating negative responses (e.g., no; No, but pre-diabetes or borderline diabetes) were grouped together *with* the addition of respondents reporting gestational diabetes only (coded as: Yes, but who were female and told only during pregnancy). Given the BRFSS defined borderline or pre-diabetes as “no” we grouped these responses with “no”, yet did run separate analyses excluding this category (data not shown) that represented less than 2% of respondents (1.6% weighted in 2015) and identified similar results as those presented in general. Thus, we decided to follow the BRFSS definition treating only those with a diagnosis of diabetes as ”yes” given diabetes was the major outcome of interest. Diabetes was treated as a dichotomous outcome for diagnoses with diabetes (excluding gestational diabetes) versus not having diabetes (including gestational diabetes). Dependent Variable 1: y = Diagnosed Diabetes (yes) versus No Diagnosed Diabetes (no).

#### 2.2.2. Dependent Variable 2

Forgone medical care due to cost (e.g., not seeking medical care that was thought to be needed due to cost barriers) among those with diagnosed diabetes was the second primary outcome of interest in the current study. The sample size was reduced when sub-setting only to those with diabetes for analyses of forgone medical care (unweighted sample size: 2011, *n* = 62,461; 2012, *n* = 59,763; 2013, *n* = 62,345; 2014, *n* = 61,118; 2015, *n* = 57,256). The survey item included, “*Was there a time in the past 12 months when you needed to see a doctor but could not because of cost?*” We subset those reporting having diabetes and who responded to this question. Thus, analyses of forgone care (yes/no) includes only those with a diagnosis of diabetes (i.e., diabetes = yes). Dependent Variable 2: Among the subset to only those saying “Yes” to having been diagnosed with diabetes, y = Yes (i.e., Yes, there was a time in the past 12 months when I needed to see a doctor but could not because of cost) versus No (i.e., No, there was not a time in the past 12 months when I needed to see a doctor but could not because of cost). 

### 2.3. Independent Variables 

Independent variables included: income, sex, education, race/ethnicity, rurality, region, and state average median household income. Because previous research has suggested that various social determinants of health such as educational attainment, low income, and racism and social exclusion, are associated with diabetes diagnoses [20,21,22], we sought to include independent variables that would capture these determinants. Further, the Framework for Action on the Social Determinants of Health also helped guide identification of individual and area-level factors related to health and related outcomes [12]. Income was coded as Don’t know/Not sure/Missing, Less than $15,000, $15,000 to less than $25,000, $25,000 to less than $35,000, $35,000 to less than $50,000, and $50,000 or more. Education was coded as did not graduate high school, graduated high school, attended college or technical school, or graduated from college or technical school. Race and ethnicity was coded as non-Hispanic White, non-Hispanic Black or African American, non-Hispanic American Indian or Alaska Native, non-Hispanic Asian, and Hispanic. “Other” was categorized as: Native Hawaiian of Other Pacific Islander, no preferred race, multiracial but preferred race not asked. For race and ethnicity, reposes for “don’t know/not sure”, or “refused” we excluded. Sex was included to assess differences across those self-reporting being male or female. Age group (coded as: 18–24; 25–34; 35–44; 45–54; 55–64; 65 and older) was included to assess diabetes and forgone care over time in descriptive analyses.

We included rurality to serve as a measure of community infrastructure and accessibility to health care resources. We separated this variable into both a 2-level and a 4-level categorization. This was defined as follows: rurality (2-level) comparing urban versus rural (not in a Metropolitan Statistical Area or MSA); rurality (4-level) comparing in the center city of an MSA, outside the center city of an MSA but inside the county containing the center city, inside a suburban county of the MSA, not in an MSA (rural). Further, U.S. Census Region was included to measure changes across larger geographic regions. States were categorized as follows: South (Delaware, District of Columbia, Florida, Georgia, Maryland, North Carolina, South Carolina, Virginia, West Virginia, Alabama, Kentucky, Mississippi, Tennessee, Arkansas, Louisiana, Oklahoma, Texas); North (Connecticut, Maine, Massachusetts, New Hampshire, Rhode Island, Vermont, New Jersey, New York, Pennsylvania); Midwest (Indiana, Illinois, Michigan, Ohio, Wisconsin, Iowa, Nebraska, Kansas, North Dakota, Minnesota, South Dakota, Missouri); Western/Pacific (Arizona, Colorado, Idaho, New Mexico, Montana, Utah, Nevada, Wyoming, Alaska, California, Hawaii, Oregon, Washington). Finally, state median household income was included to serve as a structural measure of socioeconomic status. The state average median household income for each year was included only in fully adjusted analyses. 

### 2.4. Statistical Analyses

SAS 9.4 (SAS Institute, Cary, NC, USA) was used in all statistical analyses. SAS survey procedures were used to account for the complex sampling frame of the BRFSS. SAS provides options to use survey procedures (e.g., “*proc surveylogistic*”) where survey sampling weights are included. We included SAS options for “*strata*”, “*weight*”, and “*cluster*” based on variables included in the BRFSS dataset. These survey procedures allow for weighting the data to be nationally representative on the US non-institutionalized population. Descriptive analyses were also carried out using SAS survey procedures (i.e., “*proc surveyfreq*”) to attain weighted frequencies to calculate percentages. Analyses were run using logistic regression (using “*proc surveylogistic*” in SAS) predicting (1) likelihood of being told one had diabetes; and (2) forgone medical care among those with diabetes. As mentioned, type 2 diabetes makes up approximately 95% of all cases of diabetes [2] so the implications are most specific to individuals with type 2 diabetes. To assess significant differences (at *p* < 0.01) among unadjusted bivariate comparisons with our outcomes and each independent variable, we ran a series of logistic regression models for each outcome relative to each independent variable in separate models. The fully adjusted model included several variables in each model (by year and outcome) simultaneously, where inclusion was determined based on our theoretical framework, the WHO’s Framework for Action on the Social Determinants of Health [12]. This framework closely aligns with the variables included in the fully adjusted model for both individual-level characteristics such as race, ethnicity, income, etc., and structural-level factors such as rurality and aggregate measures of income. This fully adjusted analyses included: income, sex, education, race/ethnicity, rurality, region, and state average median household income. To subset analyses for forgone medical care among those diagnosed with diabetes, “*domain*” statements in combination with survey procedures were included in the SAS code to subset to only those with diabetes to assess forgone medical care. Finally, we pooled data for all 5 years to determine difference in our 2 outcomes in bivariate and multivariable logistic regression analyses.

### 2.5. Ethics

Approval for this secondary data analysis was gained from the Texas A&M University’s Institutional Review Board in 2016 (IRB identification code: IRB2016-0712M).

## 3. Results

### 3.1. Diagnosed with Diabetes

**Figure 1:**
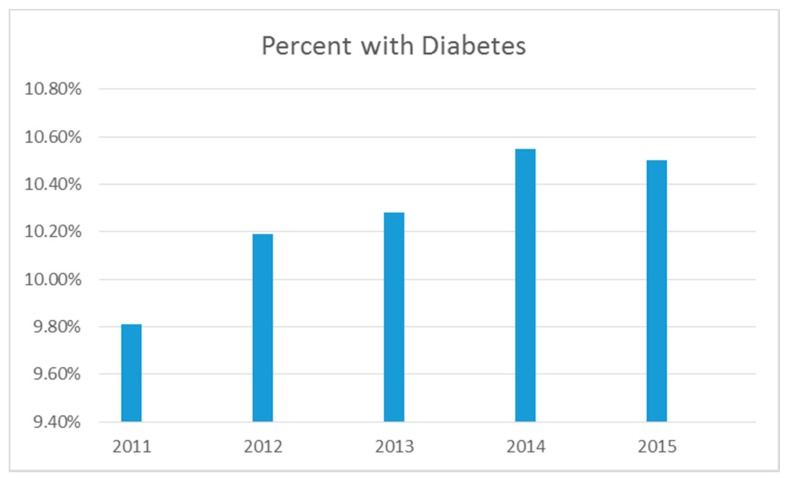
Percentage of adults with diagnosed diabetes, 2011–2015.

The prevalence rate of diabetes among adults ages 18 and older living in the U.S. was approximately 10% in 2011 and nearly 11% in 2015 (see Table 1 and Figure 1). As shown in Table 1, the prevalence of diabetes varied by age, income level, sex, education level, race/ethnicity, urbanicity, and geographic region. The rate of diagnosed diabetes increased with age. The rate was higher than 10% among those age 45 and older for every year of data included in the study. Those in the oldest age group had rates above 20% in all years of study. Individuals in lower income levels had higher rates of diabetes with rates above 10% among those with annual household incomes less than $35,000. Rates by sex were similar within 1% of each other for males and females. Rates of diabetes were higher among those with lower educational levels where rates were higher than 10% among those reporting a high school education or less. The rate of diabetes was highest among American Indian or Alaska Native adults near 15% in 2011 followed by Black or African American (13%) and Hispanic (10%) adults. The rate of diabetes remained highest among American Indian or Alaska Native adults near 17% in 2015 followed by Black or African American (14%) and Hispanic (11%) adults. Rates of diabetes among rural areas were 12% in 2011 and 15% in 2015. Rates of diabetes among urban areas were 11% in 2011 and 14% in 2015. When comparing rates by U.S. Census Region, results suggest the highest rates were in the South at approximately 11% in 2011 and approximately 12% in 2015 relative to at or lower than approximately 10% in all other regions across all years under study.

### 3.2. Forgone Medical Care among Those Diangnosed with Diabetes

**Figure 2:**
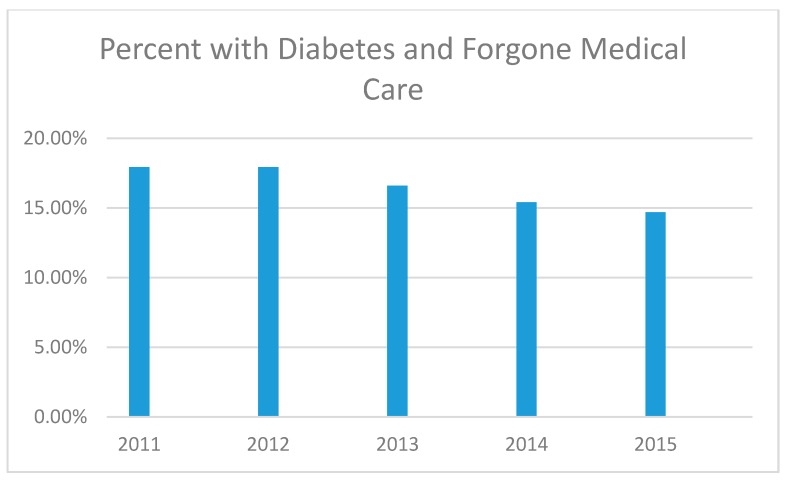
Percentage of adults with diagnosed diabetes who reported forgone medical care due to cost in the past 12 months, 2011–2015.

In total, the rate of forgone medical care among those with diabetes was nearly 18% in 2011 and nearly 15% in 2014 (see Table 1 and Figure 2). The rate of forgone medical care among those with diabetes decreased with age where those aged 18–44 making up at or near 50% for every year under study. 

The rate of forgoing medical care among those with diabetes was highest among those with lower incomes with rates over 15% for those with incomes less than $35,000. Rates of forgone medical care were higher among females with a gap of approximately 4% in 2011 and 2012 at 16% and 20% among males and females, respectively. The rates were 14% and 16% for males and females in 2015, respectively with a gap on approximately 2%. Rates of forgone medical care were higher among those with lower educations where rates were higher than 14% among those that were not college or technical school graduates. Rates were highest among those with the lowest education with rates above 20% among those who did not graduate from high school. The rate of forgone medical care was highest among Hispanic adults near 28% in 2011 followed by American Indian or Alaska Native (22%) and Black or African American (22%) adults. The rate of diabetes remained highest, albeit lower across years, among Hispanics near 23% in 2015 followed by American Indian or Alaska Native (21%) and Black or African American (18%) adults.

Forgone medical care for those living in rural areas ranged from 17% in 2011 to 13% in 2015, declining each year. Forgone medical care among urban residents ranged from 15% in 2011 to 12% in 2015. When comparing rates by U.S. Census Region, results suggest the highest rates in the South at approximately 22% in 2011 relative to rates at or less than 17% in all other regions. Rates in the South were approximately 18% in 2015 relative to at or lower than approximately 13% in all other regions.

### 3.3. Bivariate Logistic Regression for Those Diagnosed with Diabetes

Table 2 presents unadjusted (bivariate) analyses predicting the odds of diabetes. Across all years of study, the likelihood of being diagnosed with diabetes was higher (*p* < 0.01) among those with lower incomes (relative to the highest income; above $50,000). Across all years of study, the likelihood of reporting a diagnose of diabetes was higher (*p* < 0.01) among those without a college or technical degree (versus those with a college or technical school degree). Across all years of study, the likelihood of being diagnosed with diabetes was higher (*p* < 0.01) among those residing in rural areas (versus those residing in urban areas). Finally, the likelihood of being diagnosed with diabetes was higher (*p* < 0.01) in the South than all other regions. In separate analyses on pooled data we found the likelihood of being diagnosed with diabetes was lower in 2011 (OR 0.929, 99% CI 0.898–0.960) versus 2015 (*p* < 0.0001); with no differences for 2012 (OR 0.967, 99% CI 0.934–1.001), 2013 (OR 0.978, 99% CI 0.944–1.012), and 2014 (OR 1.006, 99% CI 0.973–1.041).

### 3.4. Multivariable Logistic Regression for Those Diagnosed with Diabetes 

Fully adjusted analyses included: income, sex, education, race/ethnicity, rurality, region, and state average median household income. Table 3 presents adjusted analyses predicting diabetes. Across all years of study, the likelihood of being diagnosed with diabetes was higher (*p* < 0.01) among those with lower incomes (relative to the highest income; above $50,000) after considering all other factors in the model. Across all years of study, the likelihood of being diagnosed with diabetes was higher (*p* < 0.01) among males when compared to females. Across all years of study, the likelihood of being diagnosed with diabetes was higher (*p* < 0.01) among those without a college or technical degree (versus those with a college or technical school degree). Finally, the likelihood of being diagnosed with diabetes was higher (*p* < 0.01) in the South than all other regions in 2012, 2014, and 2015 after considering all other factors in the model. In separate analyses on pooled data we found the likelihood of being diagnosed with diabetes was lower in 2011 (OR 0.693, 99% CI 0.672–0.715), 2012 (OR 0.815, 99% CI 0.788–0.843), 2013 (OR 0.896, 99% CI 0.867–0.926), and 2014 (OR 0.933, 99% CI 0.903–0.965) versus 2015 (*p* < 0.0001) after considering all other factors in the model.

### 3.5. Bivariate Logistic Regression for Forgone Medical Care among Those Diagnosed with Diabetes

Table 4 presents unadjusted analysis for forgone medical care among those diagnosed with diabetes. Across all years of study, the likelihood of forgoing medical care among those diagnosed with diabetes was higher (*p* < 0.01) among those with lower incomes (relative to the highest income; above $50,000). 

Across all years of study, the likelihood of forgoing medical care among those diagnosed with diabetes was higher (*p* < 0.01) among those without a college or technical degree (versus those with a college or technical school degree). Finally, the likelihood of forgoing medical care among those diagnosed with diabetes was higher (*p* < 0.01) in the South than all other regions.

In separate analyses on pooled data we found the likelihood of forgoing medical care among those diagnosed with diabetes was higher in 2011 (OR 1.261, 99% CI 1.148–1.385), 2012 (OR 1.267, 99% CI 1.149–1.398), and 2013 (OR 1.150, 99% CI 1.042 1.270) versus 2015 (*p* < 0.0001); with no difference for 2014 (OR 1.056, 99% CI 0.958 1.163) versus 2015.

### 3.6. Multivariable Logistic Regression for Forgone Medical Care among Those Diagnosed with Diabetes

Fully adjusted analyses included: income, sex, education, race/ethnicity, rurality, region, and state average median household income. Table 5 presents adjusted analysis for forgone medical care among those diagnosed with diabetes. Across all years of study, the likelihood of forgoing medical care among those diagnosed with diabetes was higher (*p* < 0.01) among those with annual household incomes lower than $35,000 (relative to the highest income; above $50,000). Further, the likelihood of forgoing medical care among those diagnosed with diabetes was higher (*p* < 0.01) in the South than all other regions in 2015. 

In separate analyses on pooled data we found the likelihood of forgoing medical care among those diagnosed with diabetes was higher in 2011 (OR 1.277, 99% CI 1.152–1.415) and 2012 (OR 1.191, 99% CI 1.069–1.328) versus 2015 (*p* < 0.0001); with no difference for 2013 (OR 1.018, 99% CI 0.911–1.137) and 2014 (OR 0.978, 99% CI 0.877–1.089) after considering all other factors in the model.

## 4. Discussion

The prevalence of reported diabetes for adults remained relatively stable across all years under study at 9.8% for 2011 and 10.5% for 2015, representing less than a 1% increase. There were also slight increases in the percentage with diabetes among several groups (e.g., adults with lower incomes, those who were age 65 years or older, those with who did not graduate from high school). Previous research has shown in other parts of the globe (e.g., the United Kingdom) that those in more deprived areas are more likely to be exposed to risk factors associated with diabetes [23]. Thus, relative to large increases in the rate of diabetes in the 20 years preceding 2014 (growing from 4.2 to 10%) [4], rates in the past 5 years may be plateauing to an extent, albeit still increasing slightly.

While increases in diabetes rates were small, pervasive differences remained in the current study, especially among those with lower incomes, lower levels of education, those residing in rural areas, and those in the South. Findings related to rural areas have been highlighted in previous research where residents of rural areas were found to have a higher prevalence of diabetes [24]. Of note, when considering all other terms in the model (i.e., adjusted analyses) the differences across rurality did not remain. Thus, while these difference do exist, several factors included in the model may drive these differences. Further, minority adults had consistently higher prevalence of type 2 diabetes, with American Indian or Alaska Native, Black or African American, and Hispanic adults having higher rates of diabetes than non-Hispanic White adults. Thus, the prevalence of diabetes varied by characteristics identified in the Framework for Action on the Social Determinants of Health, including geospatial factors. Further, men were more likely to have ever been diagnosed with diabetes. 

Rates of forgone medical care among adults with diabetes were highest, at 18% in 2011, but dropped under 15% in 2015. In contrast to the likelihood of ever been diagnosed with diabetes, the likelihood of forgone medical care (among those ever diagnosed with diabetes) was more likely among females. Thus, among those with a diabetes diagnosis, we find that females are particularly at-risk for forgone medical care. As with diabetes, the likelihood of forgone medical care among those diagnosed with diabetes was highly impacted by geospatial and Social Determinants of Health. Health disparities in terms of forgone medical care among those with diabetes were consistently present for those with lower incomes, those who were female, those with less education, and those residing in the South. This is consistent with earlier studies using similar data among the general population (i.e., not stratified by diagnosis or not of diabetes) when assessing forgone medical care [25]. Further, health disparities experienced over the life course may relate to social institutions and cultural differences [26]. Even more, one’s diagnosis of diabetes and subsequent health behaviors and decisions (e.g., disease management) may be impacted by economic constraints, individual priorities, and access to care among other things [26]. The fact that these factors continue to translate to disparities particularly among at-risk populations gives urgency to policy makers or other decision makers seeking ways to reduce the burden of diabetes estimated to cost $174 billion in 2007 [1] already rising to $245 billion in 2012 [27] and forgone medical care in the U.S. 

## 5. Limitations

Given data were drawn from multiple cross-sections in time, causality was not implied. Thus, only associations between our outcomes of interest and independent variables should be interpreted. The BRFSS does not specify whether individuals have a diagnosis of type 1 or type 2 diabetes. However, type 2 diabetes accounts for approximately 95% (ranging between 90% and 95%) of all diabetes [7]. Thus, while the dataset is limited in that it does not designate type 2 diabetes, it is likely representative of type 2 diabetes given the relatively high rates of type 2 versus type 1 diabetes and the fact that only adults are included and type 2 diabetes is more commonly found in adults. We were also limited in the data that could be merged with the BRFSS data using geospatial identifiers (e.g., merging 2 different datasets based on geospatial identifiers such as county). State-level characteristics not already included in the BRFSS data could be merged with the BRFSS given “state” was in the BRFSS data. However, sub-state level identifiers (e.g., county, ZIP Code, Census Tract) were suppressed in the BRFSS preventing linking more “granular” area-level characteristics (e.g., Census Tract to identify median household income at smaller geographic areas than the state). Further, self-reported surveys face the risk of recall bias, yet the major outcome was lifetime diagnosis of a specific disease and not specific recall of acute events in the recent past. Further, rates of diabetes have been shown to be lower among Alaska Native adults when compared to American Indian adults [2], yet the BRFSS grouped these individual preventing separate analyses. Additionally, the estimates in the fully-adjusted analyses are robust (i.e., *p* < 0.01). Further, while we did incorporate several factors based on theory that have been shown to be linked to our outcomes, there may be additional confounders we were not able to measure in the available data. Finally, of critical importance is that it is estimated that nearly 30% of adults with diabetes are undiagnosed and not included in the estimates of diabetes in the current study [2]. The implications of the study should be interpreted with these limitations in mind. 

### Practice and Policy Implications 

Identifying major disparities affecting millions of at-risk Americans helps to inform policy makers, decision makers, and other key stakeholders. Continued monitoring of trends in both prevalence of and factors associated with diabetes and forgone medical care among those diagnosed with diabetes can help shape policies targeted at prevention, especially targeted to the most at-risk groups (e.g., racial/ethnic adults, economically disadvantaged) and places (e.g., the South, rural areas). Knowing individual-level factors associated with risk of diabetes and forgone medical care allows for targeted diabetes screening and diabetes prevention education strategies to those most at-risk. In particular, American Indian and Alaska Native, Black or African American, and Hispanic adults are most affected by disparities. Addressing diabetes prevention among these groups takes tailored action. For example, many American Indian and Alaska Native adults reside on tribal lands that may lack resources leading to challenges for health care prevention efforts [28]. However, success has been shown with regard to translating the Diabetes Prevention Program or DPP into diverse American Indian and Alaska Native communities [28]. Another example is the evidence-based *Yo Si Puedo Controlar Mí Diabetes!* program designed as a culturally sensitive type 2 diabetes intervention for Spanish-speaking adults [29]. Thus, funding for this and similar translational efforts among diverse populations may hold hope for reaching those at most risk of developing diabetes.

While prevention of diabetes is critical, chronic disease self-management is necessary among those already diagnosed with diabetes. While there was not much change in rates over time, diabetes is chronic and health consequences are cumulative. Thus, the prevalence of diabetes builds as the population increases and as the population ages. This becomes particularly important when one thinks of the over 90% of older adults and nearly 75% of older adults who have at least one chronic condition and two or more chronic conditions in the U.S., respectively [30]. Evidence-based health and wellness programs targeting older adults suffering from chronic disease show promising outcomes (i.e., delays in illness, better disease management, and reductions in hospitalizations) [31,32,33,34]. Studies have demonstrated rural areas and other low resource areas can be reached by these programs [35], yet increased efforts seeking to understand geospatial distributions of these programs are needed to assess ongoing reach into particularly at-risk areas.

With uncertainty facing major health care programs in the U.S., particularly those programs linked to the change in federal executive and congressional leadership, it is timely to investigate the most recent trends in diabetes and access to care among those diagnosed with diabetes. This issue is even more important when considering that among the over 29 million persons affected by diabetes in the U.S., estimates suggest that nearly 8 million are undiagnosed [1,2]. Thus, while this study looks at those with a diagnosis of diabetes, more research must be done to assess factors associated with diabetes and forgone medical care among those who do not even know they have diabetes, or among those with prediabetes. Thus, efforts must be carried out to encourage potentially at-risk individuals to undergo diabetes screening in order to detect metabolic syndrome [36,37] or pre-diabetes at early stages where diabetes may be prevented [37]. Further, even after diagnosis, ongoing diabetes self-management may be effective to improve health outcomes and lessen the chance of major complications [38].

## 6. Conclusions

American Indian or Alaska Native and Black or African American adults were most at-risk for diabetes and forgone medical care among those with diabetes as compared to White non-Hispanic adults. Other major disparities were found across age, income, and education. Place-based disparities were found among those residing in rural areas and in the South. Prevention efforts and chronic disease management strategies that are tailored (e.g., culturally appropriate) to at-risk populations are needed to address both factors associated with diabetes and forgone medical care throughout the U.S.

## Figures and Tables

**Table 1:** Distribution of diabetes and forgone medical care throughout the U.S., 2011–2015.

Variables		2011		2012		2013		2014		2015	
Total Unweighted Sample Size		*n* = 506,467		*n* = 475,687		*n* = 491,773		*n* = 464,664		*n* = 441,456	
Unweighted Sample Size with Diabetes		*n* = 62,461		*n* = 59,763		*n* = 62,345		*n* = 61,118		*n* = 57,256	
		Percent with Diabetes	Percent with Diabetes and Forgone Medical Care	Percent with Diabetes	Percent with Diabetes and Forgone Medical Care	Percent with Diabetes	Percent with Diabetes and Forgone Medical Care	Percent with Diabetes	Percent with Diabetes and Forgone Medical Care	Percent with Diabetes	Percent with Diabetes and Forgone Medical Care
**Total **		9.81%	17.92%	10.19%	17.92%	10.28%	16.59%	10.55%	15.41%	10.50%	14.69%
**Age**	18–24 years	0.95%	35.06%	1.10%	34.77%	1.20%	31.27%	0.91%	22.48%	0.82%	18.19%
	25–34 years	2.18%	35.01%	2.26%	27.99%	2.10%	36.37%	2.16%	32.17%	1.80%	29.09%
	35–44 years	5.24%	29.84%	5.80%	32.80%	5.16%	26.21%	5.24%	29.54%	5.34%	23.67%
	45–54 years	10.28%	27.89%	10.52%	27.69%	10.73%	26.41%	10.97%	23.46%	10.52%	23.04%
	55–64 years	16.83%	18.83%	17.08%	20.05%	17.01%	19.34%	17.63%	16.66%	17.53%	16.36%
	65 and older	21.53%	7.11%	21.78%	6.42%	22.40%	5.91%	22.73%	6.54%	22.86%	7.35%
**Income**	Missing/don’t know	9.81%	15.59%	10.00%	16.04%	10.39%	14.11%	10.39%	13.78%	10.21%	13.10%
	<$15,000	14.98%	28.64%	15.16%	30.92%	15.68%	27.26%	16.69%	25.40%	16.39%	23.83%
	$15,000–<$25,000	12.79%	24.27%	13.24%	24.70%	13.45%	23.83%	13.85%	23.28%	14.45%	21.12%
	$25,000–<$35,000	11.46%	18.10%	11.80%	17.76%	11.82%	18.82%	11.66%	15.30%	12.03%	16.79%
	$35,000–<$50,000	9.46%	14.43%	10.32%	12.70%	10.05%	10.69%	10.41%	12.01%	10.27%	11.63%
	≥$50,000	6.51%	7.53%	6.96%	7.19%	6.91%	6.39%	7.28%	5.29%	7.48%	6.79%
**Sex**	Male	10.09%	15.70%	10.63%	15.88%	10.46%	14.79%	10.92%	13.79%	10.94%	13.50%
	Female	9.54%	20.15%	9.78%	20.01%	10.11%	18.34%	10.20%	17.05%	10.08%	15.91%
**Education**	Did not graduate High School	15.11%	23.79%	15.83%	24.36%	16.03%	23.57%	16.21%	21.91%	16.64%	20.82%
	Graduated High School	10.79%	18.33%	11.14%	18.11%	11.28%	16.54%	11.68%	15.44%	11.41%	13.86%
	Attended College or Technical School	9.12%	16.93%	9.45%	16.55%	9.61%	14.94%	9.86%	14.14%	9.88%	14.12%
	Graduated from College or Technical School	6.32%	10.41%	6.69%	10.97%	6.58%	9.46%	7.02%	9.03%	6.92%	8.98%
**Race/Ethnicity**	Hispanic	10.38%	28.33%	11.11%	27.13%	10.93%	25.84%	11.16%	23.73%	10.68%	22.83%
	Other	9.38%	21.76%	10.46%	23.67%	10.92%	19.37%	10.29%	19.78%	8.90%	19.17%
	American Indian or Alaska Native	14.76%	22.39%	14.74%	24.72%	14.76%	21.47%	15.15%	20.30%	16.68%	20.82%
	Asian	7.74%	13.00%	7.91%	14.55%	8.34%	11.67%	7.33%	13.26%	8.81%	13.86%
	Black or African American	13.22%	22.06%	13.48%	21.62%	14.23%	19.15%	14.72%	17.62%	14.32%	17.71%
	White	9.11%	14.22%	9.41%	14.02%	9.45%	13.31%	9.83%	12.31%	9.83%	11.24%
**Rurality (2-level)**	Urban	10.64%	15.23%	12.09%	14.08%	13.05%	12.14%	13.37%	11.27%	14.01%	11.44%
	Rural (Not in an MSA)	12.37%	16.93%	13.39%	15.05%	14.19%	13.91%	14.59%	13.79%	15.22%	12.75%
**Rurality (4-level)**	In the center city of an MSA	11.24%	15.85%	12.81%	14.59%	13.73%	13.41%	14.01%	11.47%	13.98%	11.03%
	Outside the center city of an MSA but inside the county containing the center city	9.80%	14.98%	11.16%	13.59%	12.39%	10.87%	12.94%	10.63%	13.50%	11.66%
	Inside a suburban county of the MSA	10.75%	14.10%	12.10%	13.64%	12.54%	10.90%	12.66%	11.73%	15.93%	13.33%
	Not in an MSA (rural)	12.37%	16.93%	13.39%	15.05%	14.19%	13.91%	14.59%	13.79%	15.22%	12.75%
**Census Region**	South	10.66%	21.27%	11.00%	21.29%	11.22%	19.47%	11.38%	18.03%	11.64%	17.84%
	North	9.46%	14.64%	9.54%	12.78%	9.75%	13.08%	10.08%	12.62%	9.59%	11.52%
	Midwest	9.46%	15.43%	9.90%	15.60%	9.69%	14.39%	10.24%	12.93%	10.08%	12.29%
	Western/Pacific	8.79%	16.50%	9.36%	18.13%	9.48%	15.93%	9.61%	15.19%	9.47%	13.46%
**State Median Income**											
**Medicaid Expansion in 2014**	Not Expanded	10.22%	19.66%	10.57%	19.48%	10.70%	17.96%	10.90%	16.68%	11.01%	16.57%
	Expanded	9.30%	15.92%	9.67%	16.33%	9.75%	15.08%	10.08%	14.09%	9.86%	12.69%

**Table 2:** Unadjusted analysis for diagnosed diabetes.

Variables		2011				2012				2013				2014				2015			
		OR	99% Confidence Intervals		*p*-Value	OR	99% Confidence Intervals		*p*-Value	OR	99% Confidence Intervals		*p*-Value	OR	99% Confidence Intervals		*p*-Value	OR	99% Confidence Intervals		*p*-Value
**Income**	Missing/don’t know	1.563 **	1.454	1.68	**<0.0001**	1.487 **	1.367	1.617	**<0.0001**	1.561 **	1.443	1.688	**<0.0001**	1.476 **	1.37	1.591	**<0.0001**	1.406 **	1.312	1.507	<0.0001
	<$15,000 vs. ≥$50,000	2.532 **	2.353	2.726		2.390 **	2.211	2.582		2.503 **	2.306	2.718		2.552 **	2.363	2.757		2.424 **	2.236	2.627	
	$15,000–<$25,000 vs. ≥$50,000	2.107 **	1.965	2.261		2.041 **	1.899	2.195		2.093 **	1.948	2.25		2.048 **	1.913	2.192		2.087 **	1.937	2.249	
	$25,000–<$35,000 vs. ≥$50,000	1.859 **	1.711	2.02		1.790 **	1.632	1.962		1.804 **	1.656	1.967		1.681 **	1.546	1.827		1.690 **	1.55	1.844	
	$35,000–<$50,000 vs. ≥$50,000	1.502 **	1.39	1.622		1.539 **	1.417	1.671		1.504 **	1.378	1.641		1.481 **	1.372	1.598		1.415 **	1.3	1.54	
**Sex**	Male vs. Female	1.064 **	1.017	1.114	**0.0005**	1.097 **	1.045	1.153	<0.0001	1.039 *	0.989	1.091	**0.0455 **	1.079 **	1.03	1.131	**<0.0001**	1.096 **	1.044	1.149	<0.0001
**Education**	Did not graduate High School vs. Graduated from College or Technical School	2.638 **	2.448	2.843	**<0.0001**	2.623 **	2.422	2.84	**<0.0001**	2.712 **	2.504	2.938	**<0.0001**	2.563 **	2.374	2.768	**<0.0001**	2.683 **	2.476	2.906	<0.0001
	Graduated High School vs. Graduated from College or Technical School	1.792 **	1.689	1.902		1.748 **	1.642	1.861		1.807 **	1.702	1.918		1.751 **	1.649	1.859		1.732 **	1.627	1.843	
	Attended College or Technical School vs. Graduated from College or Technical School	1.488 **	1.4	1.582		1.455 **	1.362	1.554		1.510 **	1.417	1.61		1.449 **	1.362	1.541		1.473 **	1.38	1.573	
**Race/Ethnicity**	Hispanic vs. White	1.156 **	1.069	1.251	**<0.0001**	1.203 **	1.109	1.306	**<0.0001**	1.175 **	1.081	1.277	**<0.0001**	1.152 **	1.067	1.245	**<0.0001**	1.097 **	1.015	1.186	<0.0001
	Other vs. White	1.033	0.882	1.209		1.125	0.955	1.325		1.174	0.993	1.389		1.052	0.918	1.206		0.897	0.777	1.034	
	American Indian or Alaska Native vs. White	1.728 **	1.448	2.061		1.665 **	1.403	1.976		1.658 **	1.385	1.985		1.639 **	1.382	1.944		1.837 **	1.542	2.188	
	Asian vs. White	0.837 *	0.689	1.017		0.827	0.644	1.063		0.872	0.704	1.08		0.726 **	0.587	0.897		0.886	0.718	1.094	
	Black or African American vs. White	1.521 **	1.416	1.632		1.500 **	1.394	1.614		1.589 **	1.477	1.71		1.584 **	1.48	1.695		1.534 **	1.428	1.647	
**Rurality**	Urban versus Rural (Not in an MSA)	0.844 **	0.802	0.887	**<0.0001**	0.890 **	0.844	0.939	**<0.0001**	0.908 **	0.860	0.958	**<0.0001**	0.904 **	0.853	0.957	**<0.0001**	0.908 **	0.853	0.965	<0.0001
**Rurality**	In the center city of an MSA versus Not in an MSA (rural)	0.898 **	0.846	0.953	<0.0001	0.951	0.889	1.017	<0.0001	0.962	0.901	1.028	<0.0001	0.954	0.892	1.021	**<0.0001**	0.906 **	0.847	0.968	<0.0001
	Outside the center city of an MSA but inside the county containing the center city versus Not in an MSA (rural)	0.770 **	0.721	0.822		0.813 **	0.758	0.872		0.855 **	0.793	0.922		0.870 **	0.803	0.944		0.870 **	0.796	0.95	
	Inside a suburban county of the MSA versus Not in an MSA (rural)	0.853 **	0.794	0.918		0.891 **	0.826	0.96		0.867 **	0.806	0.932		0.849 **	0.786	0.917		1.056	0.945	1.18	
**Census Region**	North vs. South	0.876 **	0.819	0.936	**<0.0001**	0.853 **	0.791	0.92	**<0.0001**	0.854 **	0.799	0.913	**<0.0001**	0.873 **	0.819	0.931	**<0.0001**	0.806 **	0.754	0.861	<0.0001
	Midwest vs. South	0.875 **	0.824	0.928		0.889 **	0.838	0.943		0.849 **	0.802	0.899		0.889 **	0.841	0.939		0.851 **	0.804	0.902	
	Western/Pacific vs. South	0.807 **	0.757	0.861		0.835 **	0.778	0.896		0.828 **	0.766	0.895		0.828 **	0.77	0.89		0.794 **	0.739	0.854	

** and bold indicate significantly different (alpha = 0.01); * alpha = 0.05.

**Table 3:** Adjusted analysis for diagnosed diabetes.

Variables		2011				2012				2013				2014				2015			
		OR	99% Confidence Intervals		*p*-Value	OR	99% Confidence Intervals		*p*-Value	OR	99% Confidence Intervals		*p*-Value	OR	99% Confidence Intervals		*p*-Value	OR	99% Confidence Intervals		*p*-Value
**Income**	Missing/don’t know	1.414 **	1.305	1.532	**<0.0001**	1.419 **	1.284	1.569	**<0.0001**	1.400 **	1.279	1.534	**<0.0001**	1.420 **	1.297	1.555	**<0.0001**	1.331 **	1.213	1.46	<0.0001
	<$15,000 vs. ≥$50,000	2.306 **	2.104	2.527		2.104 **	1.89	2.343		2.287 **	2.041	2.562		2.216 **	1.974	2.487		2.194 **	1.936	2.485	
	$15,000–<$25,000 vs. ≥$50,000	2.025 **	1.865	2.199		1.999 **	1.822	2.193		1.979 **	1.802	2.174		1.908 **	1.741	2.092		1.918 **	1.736	2.12	
	$25,000–<$35,000 vs. ≥$50,000	1.814 **	1.656	1.987		1.723 **	1.548	1.918		1.782 **	1.614	1.966		1.628 **	1.465	1.809		1.722 **	1.536	1.93	
	$35,000–<$50,000 vs. ≥$50,000	1.460 **	1.341	1.589		1.551 **	1.408	1.708		1.461 **	1.32	1.617		1.481 **	1.346	1.629		1.467 **	1.316	1.636	
**Sex**	Male vs. Female	1.200 **	1.14	1.263	**<0.0001**	1.302 **	1.227	1.382	**<0.0001**	1.274 **	1.201	1.351	**<0.0001**	1.283 **	1.21	1.361	**<0.0001**	1.294 **	1.214	1.38	<0.0001
**Education**	Did not graduate High School vs. Graduated from College or Technical School	1.864 **	1.702	2.041	**<0.0001**	1.771 **	1.589	1.974	**<0.0001**	1.869 **	1.68	2.08	**<0.0001**	1.890 **	1.696	2.105	**<0.0001**	1.957 **	1.737	2.204	<0.0001
	Graduated High School vs. Graduated from College or Technical School	1.342 **	1.252	1.438		1.356 **	1.25	1.471		1.408 **	1.301	1.523		1.41 **	1.303	1.525		1.411 **	1.293	1.539	
	Attended College or Technical School vs. Graduated from College or Technical School	1.298 **	1.211	1.391		1.274 **	1.173	1.384		1.331 **	1.233	1.437		1.302 **	1.204	1.407		1.344 **	1.237	1.459	
**Race/Ethnicity**	Hispanic vs. White	0.873 **	0.787	0.968	<0.0001	0.974	0.862	1.099	<0.0001	1.026	0.907	1.161	<0.0001	1.032	0.908	1.173	<0.0001	0.973	0.855	1.107	<0.0001
	Other vs. White	1.041	0.865	1.252		1.280 **	1.031	1.589		1.461 **	1.194	1.787		1.115	0.926	1.343		1.053	0.866	1.279	
	American Indian or Alaska Native vs. White	1.349 **	1.106	1.646		1.351 **	1.097	1.662		1.095	0.875	1.371		1.469 **	1.158	1.864		1.410 **	1.13	1.759	
	Asian vs. White	1.130	0.912	1.401		1.134	0.844	1.525		1.231 *	0.946	1.603		1.116	0.839	1.486		1.411 **	1.022	1.947	
	Black or African American vs. White	1.326 **	1.226	1.435		1.453 **	1.323	1.596		1.487 **	1.356	1.632		1.521 **	1.387	1.669		1.380 **	1.254	1.519	
**Rurality**	In the center city of an MSA versus Not in an MSA (rural)	0.984	0.923	1.048	0.1736	1.045	0.976	1.12	0.2898	1.036	0.967	1.108	0.5870	1.013	0.943	1.087	0.6948	1.045	0.972	1.123	0.1009
	Outside the center city of an MSA but inside the county containing the center city versus Not in an MSA (rural)	0.942 *	0.878	1.010		0.994	0.922	1.073		1.024	0.949	1.106		1.037	0.955	1.127		1.054	0.964	1.153	
	Inside a suburban county of the MSA versus Not in an MSA (rural)	0.976	0.904	1.055		1.026	0.945	1.113		1.013	0.937	1.094		1.006	0.926	1.094		1.107*	0.985	1.245	
**Census Region**	North vs. South	0.924 *	0.847	1.008	**<0.0001**	0.902 **	0.815	0.998	0.0001	0.918 *	0.840	1.003	**0.0075**	0.910 **	0.833	0.993	**0.0002**	0.840 **	0.763	0.924	<0.0001
	Midwest vs. South	0.911 **	0.854	0.973		0.926 **	0.866	0.99		0.936 *	0.873	1.003		0.900 **	0.841	0.964		0.921 **	0.857	0.991	
	Western/Pacific vs. South	0.821 **	0.759	0.888		0.864 **	0.791	0.945		0.898 **	0.814	0.991		0.901 **	0.819	0.991		0.879 **	0.795	0.973	
**State Median Income**	State Median Income (continuous variable)	1.000	1.000	1.000	0.0192	1.000	1.000	1.000	<0.0001	1.000	1.000	1.000	<0.0001	1.000	1.000	1.000	<0.0001	1.000	1.000	1.000	<0.0001

** and bold indicate significantly different (alpha = 0.01); * alpha = 0.05.

**Table 4:** Unadjusted analysis for forgone medical care among those diagnosed with diabetes.

Variables		2011				2012				2013				2014				2015			
		OR	99% Confidence Intervals		*p*-Value	OR	99% Confidence Intervals		*p*-Value	OR	99% Confidence Intervals		*p*-Value	OR	99% Confidence Intervals		*p*-Value	OR	99% Confidence Intervals		*p*-Value
**Income**	Missing/don’t know	2.267 **	1.774	2.896	**<0.0001**	2.466 **	1.810	3.359	**<0.0001**	2.408 **	1.807	3.208	**<0.0001**	2.860 **	2.209	3.702	**<0.0001**	2.069 **	1.560	2.746	<0.0001
	<$15,000 vs. ≥$50,000	4.926 **	3.978	6.099		5.777 **	4.534	7.361		5.492 **	4.222	7.143		6.094 **	4.865	7.635		4.294 **	3.260	5.657	
	$15,000–<$25,000 vs. ≥$50,000	3.933 **	3.174	4.873		4.232 **	3.321	5.391		4.585 **	3.544	5.931		5.431 **	4.372	6.746		3.675 **	2.800	4.824	
	$25,000–<$35,000 vs. ≥$50,000	2.712 **	2.097	3.506		2.786 **	2.070	3.750		3.396 **	2.518	4.580		3.234 **	2.468	4.237		2.770 **	2.042	3.758	
	$35,000–<$50,000 vs. ≥$50,000	2.069 **	1.593	2.687		1.877 **	1.407	2.502		1.753 **	1.248	2.462		2.442 **	1.881	3.171		1.807 **	1.304	2.503	
**Sex**	Male vs. Female	0.738 **	0.653	0.835	**<0.0001**	0.754 **	0.659	0.864	**<0.0001**	0.773 **	0.674	0.887	**<0.0001**	0.778 **	0.683	0.888	**<0.0001**	0.825 **	0.715	0.952	0.0005
**Education**	Did not graduate High School vs. Graduated from College or Technical School	2.685 **	2.229	3.236	**<0.0001**	2.613 **	2.108	3.241	**<0.0001**	2.952 **	2.417	3.606	**<0.0001**	2.827 **	2.297	3.481	**<0.0001**	2.664 **	2.011	3.529	<0.0001
	Graduated High School vs. Graduated from College or Technical School	1.930 **	1.625	2.293		1.794 **	1.473	2.187		1.897 **	1.578	2.281		1.84 **	1.516	2.233		1.631 **	1.256	2.116	
	Attended College or Technical School vs. Graduated from College or Technical School	1.753 **	1.471	2.089		1.610 **	1.314	1.972		1.682 **	1.389	2.037		1.661 **	1.366	2.018		1.666 **	1.275	2.178	
**Race/Ethnicity**	Hispanic vs. White	2.385 **	2.006	2.836	**<0.0001**	2.284 **	1.898	2.749	**<0.0001**	2.270 **	1.884	2.736	**<0.0001**	2.216 **	1.862	2.638	**<0.0001**	2.337 **	1.930	2.830	<0.0001
	Other vs. White	1.677 **	1.207	2.330		1.902 **	1.330	2.720		1.565 **	1.112	2.204		1.757 **	1.307	2.360		1.874 **	1.260	2.787	
	American Indian or Alaska Native vs. White	1.740 **	1.155	2.622		2.014 **	1.365	2.971		1.781 **	1.209	2.622		1.815 **	1.216	2.710		2.078 **	1.330	3.246	
	Asian vs. White	0.901	0.532	1.527		1.044	0.475	2.295		0.861	0.412	1.798		1.089	0.527	2.252		1.271	0.624	2.591	
	Black or African American vs. White	1.707 **	1.442	2.020		1.692 **	1.426	2.008		1.543 **	1.295	1.839		1.524 **	1.284	1.807		1.701 **	1.422	2.035	
**Rurality**	Urban versus Rural (Not in an MSA)	0.882 *	0.768	1.012	0.0189	0.925	0.795	1.078	0.1905	0.855 *	0.721	1.013	**0.0176**	0.794 **	0.665	0.948	0.0008	0.884	0.722	1.083	0.1177
**Rurality**	In the center city of an MSA versus Not in an MSA (rural)	0.925	0.787	1.086	0.0288	0.965	0.801	1.161	0.4140	0.958	0.778	1.18	**0.0006**	0.810 **	0.666	0.986	0.0089	0.849 *	0.691	1.043	0.1272
	Outside the center city of an MSA but inside the county containing the center city versus Not in an MSA (rural)	0.865 *	0.720	1.039		0.888	0.719	1.097		0.755 **	0.600	0.949		0.744 **	0.572	0.967		0.903	0.635	1.285	
	Inside a suburban county of the MSA versus Not in an MSA (rural)	0.806 **	0.659	0.985		0.892	0.695	1.144		0.757 **	0.608	0.943		0.831	0.649	1.063		1.053	0.749	1.480	
**Census Region**	North vs. South	0.635 **	0.530	0.761	**<0.0001**	0.542 **	0.426	0.688	**<0.0001 **	0.622 **	0.508	0.762	**<0.0001**	0.657 **	0.541	0.797	**<0.0001**	0.600 **	0.490	0.735	<0.0001
	Midwest vs. South	0.676 **	0.576	0.793		0.684 **	0.578	0.808		0.695 **	0.596	0.811		0.675 **	0.576	0.792		0.646 **	0.545	0.765	
	Western/Pacific vs. South	0.731 **	0.619	0.864		0.819 **	0.687	0.976		0.784 **	0.634	0.968		0.814 **	0.671	0.988		0.716 **	0.575	0.893	
**State Median Income**	State Median Income (continuous variable)	1.000	1.000	1.000		1.000	1.000	1.000		1.000	1.000	1.000		1.000	1.000	1.000		1.000	1.000	1.000	

** and bold indicate significantly different (alpha = 0.01); * alpha = 0.05.

**Table 5:** Adjusted analysis for forgone medical care among those diagnosed with diabetes.

Variables		2011				2012				2013				2014				2015			
		OR	99% Confidence Intervals		*p*-Value	OR	99% Confidence Intervals		*p*-Value	OR	99% Confidence Intervals		*p*-Value	OR	99% Confidence Intervals		*p*-Value	OR	99% Confidence Intervals		*p*-Value
**Income**	Missing/don’t know	1.766 **	1.327	2.351	**<0.0001**	2.327 **	1.540	3.515	**<0.0001**	1.816 **	1.225	2.694	**<0.0001**	2.156 **	1.510	3.080	**<0.0001**	1.674 **	1.115	2.514	<0.0001
	<$15,000 vs. ≥$50,000	3.608 **	2.734	4.762		4.703 **	3.274	6.757		3.869 **	2.539	5.895		4.621 **	3.157	6.764		3.256 **	2.134	4.968	
	$15,000–<$25,000 vs. ≥$50,000	3.097 **	2.379	4.031		3.760 **	2.660	5.316		3.428 **	2.318	5.069		4.310 **	3.125	5.943		2.629 **	1.819	3.801	
	$25,000–<$35,000 vs. ≥$50,000	2.397 **	1.783	3.221		2.522 **	1.739	3.657		2.842 **	1.904	4.241		2.253 **	1.539	3.297		2.220 **	1.435	3.434	
	$35,000–<$50,000 vs. ≥$50,000	1.890 **	1.398	2.556		1.710 **	1.173	2.493		1.323	0.856	2.046		2.197 **	1.518	3.181		1.376	0.863	2.195	
**Sex**	Male vs. Female	0.823 **	0.711	0.953	**0.0006**	0.793 **	0.656	0.957	0.0015	0.836 *	0.693	1.008	0.0134	0.917	0.760	1.107	0.2347	1.013	0.814	1.261	0.8751
**Education**	Did not graduate High School vs. Graduated from College or Technical School	1.214 *	0.946	1.559	0.0118	1.063	0.787	1.434	0.8511	1.320 *	0.921	1.891	0.0793	1.091	0.781	1.523	0.6820	1.427 *	0.962	2.117	0.0410
	Graduated High School vs. Graduated from College or Technical School	1.188 *	0.956	1.478		1.077	0.804	1.443		1.285	0.904	1.827		1.065	0.787	1.442		1.138	0.792	1.634	
	Attended College or Technical School vs. Graduated from College or Technical School	1.309 **	1.061	1.614		1.103	0.830	1.466		1.317 *	0.998	1.739		1.135	0.854	1.508		1.297	0.886	1.897	
**Race/Ethnicity**	Hispanic vs. White	2.001 **	1.578	2.538	**<0.0001**	1.817 **	1.329	2.485	**<0.0001**	1.846 **	1.354	2.518	**<0.0001**	1.831 **	1.346	2.491	**<0.0001**	1.826 **	1.312	2.54	<0.0001
	Other vs. White	1.673 **	1.148	2.437		1.920 **	1.186	3.107		1.679 **	1.050	2.686		1.931 **	1.240	3.009		1.623 *	0.927	2.843	
	American Indian or Alaska Native vs. White	1.577 *	0.949	2.620		1.926 **	1.204	3.081		1.501	0.871	2.587		1.867 **	1.133	3.076		1.857 **	1.032	3.343	
	Asian vs. White	1.228	0.665	2.267		1.766	0.650	4.796		1.664	0.554	4.992		1.306	0.456	3.741		2.280	0.627	8.288	
	Black or African American vs. White	1.313 **	1.085	1.589		1.338 **	1.066	1.679		1.239 *	0.994	1.543		1.284 **	1.023	1.611		1.456 **	1.113	1.905	
**Rurality**	In the center city of an MSA versus Not in an MSA (rural)	0.878	0.736	1.047	0.2051	0.934	0.769	1.133	0.6691	0.947	0.766	1.171	0.3999	0.805 *	0.646	1.003	0.0279	0.897	0.702	1.146	0.4200
	Outside the center city of an MSA but inside the county containing the center city versus Not in an MSA (rural)	0.993	0.816	1.21		1.020	0.815	1.278		0.854	0.668	1.092		0.838	0.641	1.095		0.988	0.727	1.343	
	Inside a suburban county of the MSA versus Not in an MSA (rural)	0.925	0.745	1.149		1.055	0.808	1.378		0.908	0.717	1.149		1.001	0.774	1.296		1.113	0.781	1.586	
**Census Region**	North vs. South	0.789 *	0.619	1.005	**0.0007**	0.629 **	0.459	0.862	<0.0001	0.791 *	0.585	1.070	0.0301	0.818	0.621	1.077	**<0.0001**	0.717 **	0.532	0.966	0.0010
	Midwest vs. South	0.764 **	0.633	0.920		0.766 **	0.637	0.921		0.806 **	0.660	0.985		0.695 **	0.565	0.855		0.769 **	0.608	0.973	
	Western/Pacific vs. South	0.791 **	0.635	0.985		0.832	0.647	1.069		0.838	0.603	1.163		0.754 **	0.571	0.995		0.652 **	0.438	0.971	
**State Median Income**	State Median Income (continuous variable)	1.000	1.000	1.000	0.0184	1.000	1.000	1.000	0.4139	1.000	1.000	1.000	0.0035	1.000	1.000	1.000	0.0554	1.000	1.00	1.000	0.0233

** and bold indicate significantly different (alpha = 0.01); * alpha = 0.05.
